# 2-Hydroxyisoquinoline-1,3(2H, 4H)diones (HQDs), novel inhibitors of the HIV integrase catalytic activity with a high barrier to resistance

**DOI:** 10.1186/1742-4690-10-S1-P16

**Published:** 2013-09-19

**Authors:** Frauke Christ, Jonas Demeulemeester, Belete Desimmie, Virginie Suchaud, Oliver Taltynov, Cedric Lion, Fabrice Bailly, Sergei Strelkov, Philipe Cotelle, Zeger Debyser

**Affiliations:** 1 Molecular Virology and Gene Therapy, KULeuven, Leuven, Belgium; 2 Organic and Medicinal Chemistry, University of Lille, Lille, France; 3 Laboratory of Biocrystallography, KULeuven, Leuven, Belgium

## Background

Current HIV-1 integrase inhibitors, such as raltegravir (MK-518), target the strand transfer activity of the viral enzyme HIV-1 integrase, which is vital for the HIV-1 replication process and sustained viral infection. Inhibition of integration by raltegravir is accompanied by an extremely rapid and strong reduction in viral load. However, in contrast to prior predictions based on *in vitro* experimentation, resistance evolves readily in the clinic, necessitating the efforts to develop second generation integrase inhibitors. Against this background we developed a novel class of INSTIs, the 2-hydroxyisoquinoline-1,3(2H, 4H)diones (HQDs).

## Methods

We have performed detailed analysis of the inhibition of integration by HQDs. Mechanistic studies (TOA and QPCR) and combination experiments shed light on the competiveness of HQDs with known inhibitors of HIV replication including INSTIs. Cross-resistance profiling, resistance selection and co-crystallization with the PFV-intasome allow detailed understanding of the underlying mechanism of anti-HIV activity.

## Results

Biochemical evaluation of HQD demonstrates that this novel class of catalytic site inhibitors potently blocks both the 3’ processing and strand transfer reaction of integrase. TOA and QPCR demonstrate that indeed HQDs target the integration step of HIV-replication in cell culture. Interestingly HQDs place the barrier to resistance very high, resulting in failure to select for genotypically resistant HIV strains (even after more than 100 passages under selective pressure). A high resolution (3.4 Å) structure of HDQ bound in the catalytic site of the PFV-intasome provides evidence for the high barrier to resistance and allows for structure based optimization of HQDs’ antiviral activity. Preliminary ADMETox profiling demonstrates that HQDs are associated with low cellular toxicity.

## Conclusion

HQDs are a novel class of integration inhibitors with a beneficial high barrier to resistance development. The detailed structural analysis of HQDs bound in the PFV-intasome active site allows improvement of the antiviral activity from the sub-micromolar range towards nanomolar activities which will pave the way for further pre-clinical development of these novel small molecule catalytic site inhibitors of HIV-integration.

